# Receipt of specialized palliative care and health care utilization at the end of life in hematological cancer patients – the Stockholm experience

**DOI:** 10.2340/1651-226X.2025.42189

**Published:** 2025-02-10

**Authors:** Lena von Bahr, Peter Strang, Torbjörn Schultz, Per Fürst

**Affiliations:** a Department of Medicine, Sahlgrenska Academy, Gothenburg University, Sweden; b Section of hematology and coagulation, Sahlgrenska University Hospital, Gothenburg, Sweden; c Department of Oncology-Pathology, Karolinska Institutet, Stockholm, Sweden; d Research Department Palliative Care, Stockholm Sjukhem Foundation, Stockholm, Sweden; e Department of Neurobiology, Care Sciences and Society, Karolinska Institute, Stockholm, Sweden

**Keywords:** Observational study, oncology, health economics, terminal care, emergency room visits, health resources, quality indicators/health care

## Abstract

**Background:**

The treatments of hematological malignancies tend to be intense, and compared with solid tumors, less is known about the health care consumption during end of life (EOL). Therefore, the aim was to study the receipt of specialized palliative care (SPC) and how it affects health care utilization, in relation to sex, age, socioeconomics, and frailty risk (Hospital Frailty Risk Score [HFRS]).

**Methods:**

In a retrospective, observational registry study, all patients who died of a hematological malignancy during the years 2015–2021 in the Stockholm County were included and analyzed with descriptive statistics and logistic regression models.

**Results:**

Of the 2,858 included patients (mean age 76 years, 41% women), 38% had myeloid malignancies, 41% lymphocytic malignancies, and 21% had myeloma. During the last 3 months of life, 56% received SPC, with an overrepresentation of women, aOR 1.35 (1.16–1.58, *p* < 0.0001), whereas persons with risk of frailty (HFRS) were underrepresented, aOR 0.74 (0.63–0.86, *p* < 0.0001). Unplanned ER visits were more likely in persons aged over 80 years (*p* = 0.004) and in persons with frailty risk (*p* < 0.0001). Patients receiving SPC had a substantially reduced likelihood of ER visits, aOR 0.34 (0.29–0.40, *p* < 0.0001). Emergency hospitals as place of death was positively associated with frailty risk, aOR 1.50 (1.23–1.83, *p* < 0.0001) but negatively associated with age over 80 years (*p* < 0.0001) and especially with receipt of SPC, aOR 0.05 (0.04–0.06, *p* < 0.0001).

**Interpretation:**

Receipt of SPC could possibly reduce the need for emergency care in the end of life and the Stockholm model might facilitate referral to SPC for hematological patients.

## Introduction

Hematological malignancies comprise about 8% of all new cancer cases, and they are the cause of death in about 2,000 patients per year in Sweden [1, 2]. This is likely to increase in coming years due to an aging population, which stresses the importance of effective utilization of health care resources. Health care utilization naturally increases as we approach end of life (EOL) [3], and it is of utmost importance that the quality and nature of the health care align with the needs and wishes of patients. Intensive treatments can drastically impact the quality of life during the last days and months [4], and ER visits and ICU admissions during the last 30 days of life are adopted as negative quality measures of oncology care, including hemato-oncology [5].

Patients with hematological malignancies have a lower receipt of palliative care than other cancer patients [6, 7], and their last months are commonly spent in a hospital undergoing intensive treatments [4, 6, 8]. The panorama of hematological malignancies has many facets. Aggressive diagnoses like acute leukemia and high-grade lymphomas have been described as ‘rollercoasters’ [9], characterized by unpredictable disease courses where complete remissions with long-term survival are sometimes achieved even at the brink of death. In multiple myeloma, while so far being an incurable disease, a range of new treatments have been approved over the last decade [10] reporting complete responses in heavily treated, multiply refractory patients [11, 12]. This sets the scene for a situation where both health providers and patients keep pushing treatments, sometimes at the expense of quality of life [13, 14]. Though modern palliative care is moving towards earlier integration [9, 15, 16], it is still often perceived as equivalent to end-of-life care [17] and is therefore shunned, as hope for a cure remains.

In Stockholm County, Sweden, with 2.4 million inhabitants, Region Stockholm has the financial and practical responsibility for all healthcare services, including specialized palliative care (SPC), while the municipalities have the financial responsibility for nursing homes. In the case of SPC, changes in organization and financial reimbursement were made around 2012 and the Region can since then either carry out the care themselves or contract external palliative care services who carry out SPC on behalf of the Region. As a result, more patients, including hematological patients, were admitted to SPC services. These changes mean that teams specializing in palliative care for both cancer and other chronic diseases are increasingly also providing supportive care at home to hematological patients during both curative and palliative treatments, and where necessary, specialized inpatient palliative care units are available. Consequences of this increasingly applied and appreciated service are to enable patients to stay at home during treatment while maintaining medical safety. The teams provide round-the-clock service including transfusions, parenteral nutrition, and intravenous antibiotics at home. Although the introduction of palliative care earlier than before was not the aim of the changes, they are leading to many hematology patients encountering palliative care teams earlier than before during their treatment, which can facilitate the transition to palliative care if needed.

The aim of this paper was to study SPC receipt and how it affects the health care utilization at EOL for patients with hematological malignancies in the Stockholm region, as hematological care differs from cancer care as regards use of health care services. We explored possible differences in the receipt of SPC, visits to the emergency room, and acute hospital as well as place of death (PoD) in relation to hematological diagnosis age, sex, receipt of SPC, socioeconomic status (SES), and risk of frailty.

## Materials and methods

The Strengthening the Reporting of Observational Studies in Epidemiology (STROBE) criteria have been used to report the methods and result.

### Study design

This retrospective observational registry data study was mainly based on Stockholm Region’s central data warehouse (the VAL database) for administrative data. In the Stockholm Region, reporting of any care consumption is mandatory and is a basis for economical compensation for the units. The reported data are close to 100%, that is with extremely few missing values. Some of the data are explicitly reported, for instance sex, ICD-10 codes, number of out care visits, length of stay, etc. Other variables, such as Hospital Frailty Risk Score (HFRS), are calculated from ICD-10 data.

Data were collected for patients who died during the seven consecutive years, 2015–2021, with a diagnosis of hematological malignancy (ICD-10 C81–C96 + D45–D47). This time frame was chosen as it was the most recent with available data and to arrive at a sufficiently large number of patients per diagnosis group to enable reliable regression analysis. There were no tangible changes in the organization of SPC in the region during this time period. The analysis concerned various aspects including sex, age, receiving SPC, SES, and risk of frailty, chosen to assess equality of care and factors of known impact on SPC receipt [18].

In the Stockholm Region, reporting of any care consumption is mandatory and is a basis for the economical compensation for the units. The reported data are close to 100%, that is with extremely few missing values. Some of the data are explicitly reported, for instance sex, ICD-10 codes, number of out care visits, length of stay, etc. Other variables, such as HFRS, are calculated from ICD-10 data.

### Population

This study included patients aged ≥ 18 years who died in Stockholm during the 7 consecutive years, 2015–2021, with a diagnosis of hematological malignancy and who did not reside in a nursing home.

Patients in nursing homes were excluded, since care in nursing homes in Sweden is mainly the responsibility of the communities, whereas all other forms of care are the duty of the county councils (nowadays called ‘Regions’ according to the new terminology in Sweden). By the agreements between Region and Municipality, patients cannot receive SPC while in a nursing home.

The hematological diagnoses were grouped as follows: Lymphocytic malignancies (including lymphomas, chronic lymphocytic leukemia, and acute lymphocytic leukemia): ICD-10 C81–C88 + C91. Myelomas: ICD-10 C90. Myeloid malignancies (including acute myeloid leukemia, myelodysplastic syndrome, and myeloproliferative neoplasms): ICD-10 C92–95 + D45–47. The grouping reflects the organization of hematological cancer care in Karolinska University Hospital, Stockholm, the main tertiary referral center caring for hematological patients in Stockholm County.

## Variables

Receipt of SPC during the last 3 months, Emergency Department (ED) visits during the last month, as well as acute hospital as PoD, were outcome measures. Even though this study focused on the last month of life, the receipt of SPC refers to the last 3 months of life. This was done to improve the identification of the right patient group and not just individual palliative care interventions, as this is based on ICD codes. Emergency hospital did not include geriatric clinics located in emergency hospitals or separate geriatric hospitals. Age, sex, receiving SPC, socioeconomic Mosaic groups on an area level (not individual level), HFRS, and diagnosis (myeloma, lymphocytic malignancies, myeloid malignancies) were used as explanatory variables.

Mosaic is a system that divides a county or city into socio-economic areas [19, 20]. Stockholm County is divided into 1,300 small areas, and each area is classified as Mosaic 1, 2, or 3, where Mosaic Group 1 corresponds to the most affluent areas. The three groups are of approximately the same size. Mosaic provides socioeconomic information, and based on this, the Stockholm Regional Council can define and distribute different housing areas to the three different socioeconomic classes. This area-based SES is a broad measure of SES that includes classical variables, such as income and education, as well as additional variables, such as lifestyle, cultural aspects, and living arrangements. Therefore, mosaic groups may be less specific than traditional SES measures, but SES measures on an individual level were not available through the VAL database. Overall, cluster analyses of more than 40 iterative variables form the basis for the mosaic groups. The initial analyses included all the mosaic groups. However, the data were finally dichotomized based on the data distribution; Mosaic 1 and 2 (i.e. affluent and middle-class areas) were merged and compared with Mosaic 3 as Mosaic groups 1 and 2 have shown obvious similarities in recent studies, as opposed to Mosaic group 3, the least advantaged group [18, 21, 22].

HFRS is a measure of the risk of frailty, based on 109 weighted ICD-10 diagnoses instead of the collection of data prospectively through patient- or staff-administered frailty instruments [23]. HFRS was developed based on a development cohort of over 22,000 patients with frailty and validated in a cohort of over 1 million patients, including patients with cancer. According to Reference [23], patients with HFRS values of <5 are judged to have a low frailty risk (non-frail), patients with values between 5 and 15 have an intermediate risk, whereas people with values above 15 are judged to constitute a high-risk group. This is the definition applied throughout the following analysis.

### Selection bias

#### Missing data

Each clinic and care unit must report to the VAL, in most cases, even as a basis for their remuneration, which means that the data are close to complete with few missing values. Each person who has used public healthcare during the years studied is included in the VAL database, which also includes most forms of private care as private care providers have economic agreements with the regional council.

### Study size

Since all the deaths from hematological malignancies that occurred between 2015 and 2021 were included in the study, no power calculations were performed.

### Statistical methods and missing data

Proportions were compared using t-tests and chi-square tests. There were few missing data (mainly the Mosaic classification, in 13 patients; therefore, these 13 patients were excluded from the analyses). Regarding clinically relevant variables, univariable logistic regression models were performed, followed by multivariable logistic regression models. The SAS 9.4/Enterprise guide 8.2 was used for the statistical analysis.

### Ethics

The Regional Ethical Review Authority (EPN 2017/1141-31, 2023/03378-01) approved this study.

## Results

### Clinical characteristics

During 2015–2021, 3,425 people older than 18 years died from hematological malignancies in the Stockholm County. Of these, 2,858 did not reside in nursing homes and formed the main study group (13 patients with missing mosaic group were excluded). Forty-one per cent were women, 593 (21%) had been diagnosed with myeloma, 1,169 (41%) with lymphocytic malignancies, and 1,096 (38%) with myeloid malignancies. The distribution of hematological malignancies in the nursing home patients did not differ significantly from the study population.

The mean age for the main study group was 76 years, and there were no significant differences in age between the three hematological subgroups.

According to the HFRS scores, 1,507 (53%) patients were classified as having an increased risk of frailty. Patients with myeloma were more often risking frailty (61%) than patients with lymphocytic (50%) or myeloid malignancies (51%), (*p* < 0.0001). About half of the patients were admitted to an emergency hospital at least once during their last month of life, patients with myeloid malignancies somewhat more seldom (51%) than myeloma (55%) and lymphocytic malignancies (57%), (*p* = 0.01). On the other hand, patients with myeloid malignancies were more often admitted to a hematology department (17%) than patients with myeloma (12%) or lymphocytic malignancies (16%) (*p* 0.05) (Table 1).

**Table 1:** Demographic and clinical data (*n* = 2858).

Variable	Total *N* = 2858	Myeloma *n* = 593	Lymphocytic malignancies *n* = 1169	Myeloid malignancies *n* = 1096	*p*
**Age**					
Mean, years (SD)	76.0 (12)	76.5 (10)	76.0 (12)	75.6 (13)	0.82
Women	76.6 (12)	77.4 (10)	76.3 (12)	76.5 (14)	0.57
Men	75.5 (12)	75.8 (10)	75.9 (11)	75.0 (13)	0.81
**Sex**					0.01
Women (%)	1165/2858 (41)	260/593 (44)	439/1169 (38)	467/1096 (43)	
**Mosaic**					0.43
Group 1+2 (%)	1966/2858 (78)	419/593 (71)	806/1169 (69)	741/1096 (68)	
**Frailty score**					< 0.0001
Linear (SD)	6.6 (5.4)	7.5 (5.8)	6.4 (5.3)	6.3 (5.4)	
**Frailty score**					< 0.0001
Groups 2+3 *n* (%)	1507/2858 (53)	362/593 (61)	588/1169 (50)	557/1096 (51)	
**Emergency department visit ≥ once**					0.03
Last month of life *n* (%)	1498/2858 (52)	326/593 (55)	631/1169 (54)	541/1096 (49)	
Admissions to emergency hospital*					0.01
Last month of life *n* (%)	1563/2858 (55)	329/593 (55)	672/1169 (57)	562/1096 (51)	
**Died in emergency hospitals (%)**	1040/2858 (36)	204/593 (34)	424/1169 (36)	412/1096 (38)	0.43
**Admitted to hematology department**					0.05
Last month of life *n* (%)	440/2858 (15)	73/593 (12)	184/1169 (16)	183/1096 (17)	
**Died in a hematology department**					0.0009
*n* (%)	315/2858 (11)	49/593 (8)	116/1169 (10)	150/1096 (14)	
**Received SPC**					0.90
Last 3 months of life *n* (%)	1596/2858 (56)	336/593 (57)	650/1169 (56)	610/1096 (56)	
**SPC Place of Death**					0.98
*n* (%)	1470/2858 (51)	306/593 (52)	599/1169 (51)	565/1096 (52)	

**Mosaic** provides socioeconomic information on an area level. Groups 1 and 2 correspond to persons from the two most affluent areas and Group 3 from the least affluent.

**Frailty groups:** Group 1 ≤ 5 points (low risk of frailty), group 2 = 5–15 points (intermediate risk), group 3 ≥ 15 points (high risk).

**SPC**, specialized palliative home care and specialized palliative inpatient care.

* at least once during the last month of life.

### Receipt of specialized palliative care

Of the 2,858 patients in the main study group, a total of 1,596 (56%) had received SPC at some point during their last 3 months of life, with no significant difference seen between the hematological subgroups (Table 1).

To control the receipt of SPC for diagnosis, sex, SES, age, and risk of frailty, a multivariable logistic regression was performed. No difference in receipt of SPC was seen between the hematological subgroups, for age or for SES. Female sex increased the likelihood of receiving SPC, with an adjusted OR (aOR) of 1.35 (1.16–1.58, *p* = 0.0001). Patients with an intermediate to high risk of frailty had a reduced likelihood of receiving SPC, aOR 0.74 (0.63–0.86, *p <* 0.0001) (Table 2).

**Table 2:** Factors associated with receipt of SPC during the last 3 months of life, among patients with hematological malignancies (*n* = 2,858).

Variable	Multivariable analysis	
	OR (95% CI)	*p*
Hematologic diagnosis		
Myeloma	1.07 (0.88–1.32)	0.49
Lymphocytic malignancy	1.01 (0.86–1.20)	0.87
Myeloid malignancy	Ref.	
Sex		
Women	1.35 (1.16–1.58)	0.0001
Men	Ref.	
Age groups		
80 years or more	1.21 (1.00–1.47)	0.06
70–79 years	1.04 (0.85–1.27)	0.69
18–69 years	Ref.	
Mosaic		
3	0.96 (0.81–1.12)	0.57
1+2	Ref.	
HFRS Frailty risk groups		
2–3 (intermediate – high risk)	0.74 (0.63–0.86)	< 0.0001
1 (low risk)	Ref.	

### Emergency department visits during the last month of life

In total, 1,498 (52%) patients made one or more unplanned visits to the ED. Forty-nine per cent of patients with myeloid malignancies, 55% of patients with myeloma, and 54% of patients with lymphocytic malignancies (54%) visited the ER at least once (*p* = 0.03) (Table 1).

Accordingly, in an adjusted multiple variable logistic regression, lymphocytic malignancy was associated with more ER visits, aOR 1.23 (1.04–1.47, *p* = 0.02). Age over 80 years, aOR 1.35 (1.10–1.65, *p* = 0.004), and increased risk of frailty, aOR 1.62 (1.39–1.90, *p* < 0.0001) were also associated with more ER visits. Receiving SPC was strongly associated with a lower likelihood of visiting an ED, aOR 0.34 (0.29–0.40, *p* < 0.0001). No difference was seen regarding sex, age below 80 years or SES (Table 3).

**Table 3:** Factors associated with emergency department visits during the last month of life, among patients with hematologic malignancies (*n* = 2,858).

Variable	Multivariable analysis	
	OR (95% CI)	*p*
Hematologic diagnosis		
Myeloma	1.23 (1.00–1.52)	0.05
Lymphocytic malignancy	1.23 (1.04–1.47)	0.02
Myeloid malignancy	Ref.	
Receipt of SPC		
Yes	0.34 (0.29–0.40)	< 0.0001
No		
Sex		
Women	1.09 (0.93–1.27)	0.29
Men	Ref.	
Age groups		
80 years or more	1.35 (1.10–1.65)	0.004
70–79 years	1.17 (0.95–1.44)	0.14
18–69 years	Ref.	
Mosaic		
3	1.04 (0.88–1.23)	0.63
1+2		
HFRS Frailty risk groups		
2–3 (intermediate – high risk)	1.62 (1.39–1.90)	< 0.0001
1 (low risk)	Ref.	

### Emergency hospital as place of death

The factor most strongly associated with not dying in an emergency hospital was receipt of SPC, with an aOR 0.05 (0.04–0.06, *p* < 0.0001) for emergency hospital as PoD.

In total, slightly more than a third (36%) of the patients with a hematological malignancy died in an emergency hospital setting, with no significant difference seen between patients with myeloma, lymphocytic or myeloid malignancies (Table 1). Eleven per cent died in the hematology department (myelomas 8%, lymphocytic malignancies 10%, and myeloid malignancies 14%, *p* = 0.0009) (Table 1).

In a multivariable analysis, adjusting for sex, socioeconomic Mosaic group, age, risk of frailty, and access to palliative care, neither of the hematological subgroups were significantly associated with having an emergency hospital as PoD.

Older patients with a hematologic diagnosis were less likely to have emergency hospital as PoD, if 70–79 years old, aOR 0.71 (0.55–0.92, *p* = 0.01), if over 80 years old, aOR 0.37 (0.29–0.48, *p* < 0.0001). Increased risk of frailty (HFRS) meant a higher risk of dying in hospital, aOR 1.50 (1.23–1.83), *p* < 0.0001) (Table 4).

**Table 4:** Factors associated with having an emergency hospital as place of death among patients with hematologic malignancies (*n* = 2,858).

Variable	Multivariable analysis	
	OR (95% CI)	*p*
Hematologic diagnosis		
Myeloma	0.80 (0.61–1.04)	0.10
Lymphocytic malignancy	0.92 (0.74–1.15)	0.46
Myeloid malignancy	Ref.	
Receipt of SPC		
Yes	0.05 (0.04–0.06)	< 0.0001
No	Ref.	
Sex		
Women	1.02 (0.84–1.25)	0.83
Men	Ref.	
Age groups		
80 years or more	0.37 (0.29–0.48)	< 0.0001
70–79 years	0.71 (0.55–0.92)	0.01
18–69 years	Ref.	
Mosaic		
3	0.87 (0.71–1.07)	0.20
1+2	Ref.	
HFRS Frailty risk groups		
2–3 (intermediate – high risk)	1.50 (1.23–1.83)	< 0.0001
1 (low risk)	Ref.	

HFRS: Hospital Frailty Risk Score.

## Discussion

We examined the health care consumption and possible differences in the receipt of SPC, number of ED visits, and acute hospitals as the PoD for patients who died in the Stockholm region from hematological malignancies. In the 7-year cohort of 2,858 persons, a little more than half (56%) received SPC, which is higher than previously reported in a meta-analysis including data from the US, UK, Hong Kong, and Australia [7] as well as a recent large, nation-wide study from Germany [6] but matches that in Medicare recipients in the US [8]. The populations analyzed in these studies are relatively comparable to our study population, except for the exclusion of nursing home residents. Studies included in the meta-analysis are covering an earlier time period, some as early as the 1980s, but the German study is based on deaths from 2016 to 2020 and thus contemporary to this study. The German health care system is also relatively similar to the Swedish universal health care and thus probably the best comparator.

About a third (36%) died in an emergency hospital, with numerically small differences among the three hematological groups. The Stockholm region here again matches Medicare recipients [8], but this seems restricted to the region. Unpublished data from the Swedish Register of Palliative Care show that at least 60% of hematological patients outside of Stockholm died in an emergency hospital, in line with a Dutch study in younger hematological patients [24].

These differences are likely to be related to the organization of SPC in the Stockholm Region, leading to a high availability of SPC and lower thresholds for referring to SPC for patients already receiving supportive care via the SPC teams. It demonstrates a feasibility to expand SPC receipt for patients with hematological malignancies despite the known barriers [13, 17], and we hope that these results can be of use in planning health care organization regarding early integration of palliative care services in hematological malignancies.

The hematological diagnosis groups myeloma and lymphocytic and myeloid malignancies differ in clinical presentation and trajectory [9]. Myeloma and low-grade lymphomas are incurable diseases where treatment periods are interjected with periods of remission, but over a period of years, there can be a gradual deterioration with disease progression in parallel with complications such as kidney failure [25, 26]. Acute leukemias and high-grade lymphomas, on the other hand, present with acute onset, where intensive treatment carrying severe side effects might lead either to long-term survival or death [27, 28]. They are usually monitored closely and admitted directly to the hematology department in case of deterioration. It is therefore somewhat surprising that apart from the higher chance of dying in the hematology department for patients with myeloid malignancies, there were only minor differences between the diagnoses, taking the multiple analyses into account. This does, however, align with previous reports [8] and might reflect that clinician traditions and preferences have a larger impact than disease particularities.

Internationally, patients with hematological malignancies have a lower receipt of SPC compared to patients with solid tumors [6, 7]; they have higher rates of inpatient and intensive care and are more likely to die in a hospital [8, 29]. It is known that an early start of palliative care alongside hematological care even with a curative intent, including advance care planning, can avoid inappropriate care at the EOL [30, 31]. Our results suggest that receipt of SPC for hematological patients is associated with fewer unplanned visits to the ED and fewer deaths in the acute hospital settings. This is in line with similar data on cancer as well as on non-malignant diseases such as heart failure, COPD, ALS, and dementia [21, 22, 32].

Home palliative care is also associated with cost savings, compared to usual care [33]. However, clinical cost studies have methodological difficulties, as those in need of home care services may differ from others, as regards care needs. For example, in Region Stockholm (formerly: Stockholm County Council), only patients with complex needs and with high symptom intensity are admitted to SPC, that is a group with higher needs.

Women were more likely to receive SPC, while having an increased risk of frailty actually meant receiving SPC at a lower rate, the latter in line with what we previously reported in solid cancer [18]. That male patients are slightly less likely than female patients to receive SPC has been reported from Ontario Canada and seen in our previous research [18, 34, 35]. The reason for this is unclear.

Why patients with an increased risk of frailty have a lower receipt of SPC is also unclear, but possible reasons may be structural, that is SPC mainly admits individuals with clear and delimited assignments both in terms of scope and expected remaining time, whereas frailty is often caused by multiple diseases, or possibly by side effects of intensive hematological treatment. It should be noted that frailty in this study was defined by HFRS, a risk score composed by ICD codes for comorbidities [23] and would assign a high score to severely ill patients with treatment complications such as sepsis and kidney failure. This might also explain why the patients with high HFRS were more likely to die in the hospital.

Older patients were more likely to visit the ED during the last month of life but less likely to die in the emergency hospital. This might reflect that the decision to transition to end-of life care is taken earlier in the older patients, allowing time to transfer out of the hospital setting.

This study is based on a large dataset covering all patients dying with hematological malignancies in the Stockholm region, with 2.4 million inhabitants, and has minimal missing data, providing a robust and complete picture of the health care use during the period of study. The exclusion of nursing home residents does limit the generalizability of the results for this particular population.

A common limitation in studies addressing end-of-life in hematological malignancies is that it is unclear whether there was a possibility of cure or long-term remission, justifying the aggressive treatment. We do not have data on decisions to transition to end-of-life care or specifics about the aim of treatment. However, it is a fact that all patients in the study did die, and the uncertainty of prognosis is one of the reasons the quality of palliative care is lacking in patients with hematological malignancies [13]. There were also no major differences between myeloid and lymphocytic malignancies (possibly curable) and myelomas (incurable). It would be valuable to further study the relationship between earlier SPC access, both in curative and palliative settings, and end-of-life outcomes. New outcome measures regarding end-of-life in hematological malignancies, better capturing the individual illness trajectory and patient preferences, are also needed.

The study was not designed to evaluate QoL in the EOL. A Cochrane meta-analysis indicate that receipt of home SPC is associated with improved QoL [36], and our own data show that both cancer patients and their families are very satisfied with the symptom relief and support provided by SPC [37]. However, we plan to follow up with prospective studies in the home care setting of hematological patients treated with curative intent, with a focus on QoL for patients and caregivers.

In conclusion, we show that receipt of SPC strongly correlates with a reduced likelihood of visits to the ED and of dying in an emergency hospital for patients with hematological malignancies. We also found high proportions of SPC receipt and low proportions of death in emergency hospital in Stockholm, both in international comparisons and compared to the rest of Sweden. This suggests that the Stockholm model of broad early access to home care via SPC teams does facilitate the transition to high-quality palliative care, but this relationship needs to be further explored as no causal relationship can be concluded by this observational study.

## Data Availability

The data contain potentially identifying information regarding individuals and therefore are subject to ethical and legal restriction to public sharing. We cannot share the data set as it is not permitted by Swedish law and the ethical permission obtained only allows for public sharing on a group level.
